# Immune exhaustion in bacterial infections: mechanisms, consequences, and therapeutic implications

**DOI:** 10.1016/j.bjid.2026.105809

**Published:** 2026-04-03

**Authors:** Saeed Hemati, Zeinab Mohsenipour

**Affiliations:** a Ilam University of Medical Sciences, Faculty of Medicine, Department of Microbiology, Ilam, , Iran; b Tehran University of Medical Sciences, School of Medicine, Department of Microbiology, Tehran, Iran

**Keywords:** Bacterial infections, Immune response, Immunotherapy, Regulatory T-cells, T-cell exhaustion

## Abstract

T-cell exhaustion, a well-characterized phenomenon, has historically been studied in the context of viral and oncological diseases. However, its relevance to chronic bacterial infections has only recently garnered attention. This review summarizes emerging evidence suggesting that bacterial pathogens can induce immune exhaustion through a variety of mechanisms. Additionally, we explore how bacterial biofilms, immune-privileged niches, and regulatory T-cell expansion contribute to persistent immune dysfunction. The article further examines the consequences of immune exhaustion, including secondary infections, antibiotic resistance, and microbiome dysbiosis, which are often underappreciated aspects of chronic immune impairment. Therapeutic strategies targeting these exhaustion pathways, such as immune checkpoint blockade, metabolic reprogramming, and microbiome modulation, are also discussed. We emphasize the need to consider chronic bacterial infections not as static conditions but as dynamic processes that interact with and suppress the immune system. Thus, understanding the mechanisms behind immune exhaustion highlights the importance of developing therapies that restore immune function, rather than solely relying on traditional antimicrobial treatments.

## Highlights

• Bacterial pathogens can drive immune exhaustion, a concept traditionally studied in viral and oncologic contexts but increasingly recognized in chronic bacterial infections.
• Biofilms, immune-privileged niches, and regulatory T-cell expansion are major contributors to persistent immune dysfunction, creating barriers to effective immune responses.
• Immune exhaustion exacerbates secondary infections, antibiotic resistance, and microbiome dysbiosis, underscoring its broader impact beyond direct bacterial persistence.
• Targeting exhaustion pathways through immune checkpoint blockade, metabolic reprogramming, and microbiome modulation offers promising therapeutic strategies beyond conventional antimicrobial approaches.

## Introduction

Immune exhaustion refers to the progressive decline in immune cell functionality; a phenomenon initially studied in chronic viral infections and cancer. However, it has also been recognized in bacterial infections.^1^ T-cell exhaustion, first described by Moskophidis et al. in 1993, has predominantly been associated with CD8+ *T*-cell responses, although research has also shown that CD4+ *T*-cells can become functionally unresponsive in various chronic infections.^2^ Exhausted T-cells follow a differentiation pathway that includes progenitor exhausted T-cells, which retain some proliferative capacity and responsiveness to Immune Checkpoint Blockade (ICB), and terminally exhausted T-cells, which exhibit limited functionality and resistance to reactivation.^3^ T-cell exhaustion is driven by persistent antigen stimulation, transcriptional reprogramming, and metabolic dysfunction, particularly mitochondrial impairment and oxidative stress.^4^^,^^5^

Moreover, bacterial biofilms and antigen persistence within immune-privileged niches (e.g., the central nervous system and eyes) contribute to T-cell exhaustion.^6^^,^^7^ Exhausted T-cells are characterized by low proliferation in response to antigen-mediated T-Cell Receptor (TCR) stimulation, defects in routine functions (such as cytokine production and cytotoxic activity), impaired differentiation into memory cells, metabolic alterations (from oxidative phosphorylation to glycolysis), and the expression of high levels of inhibitory molecules such as Programmed Death-1 (PD-1), Cytotoxic T-Lymphocyte-Associated Antigen-4 (CTLA-4), T-cell Immunoglobulin domain and Mucin domain-3 (TIM-3), and Lymphocyte Activation Gene-3 (LAG-3). These markers are mainly associated with altered transcriptional programs (e.g., NR4a and TOX transcription factors) and epigenetic regulation.^8^

Therefore, this review aims to elucidate the immunological mechanisms underlying T-cell exhaustion in bacterial infections and discuss emerging therapeutic strategies aimed at restoring immune function.

## Mechanisms of immune exhaustion in different types of bacterial infections

**Fig. 1:**
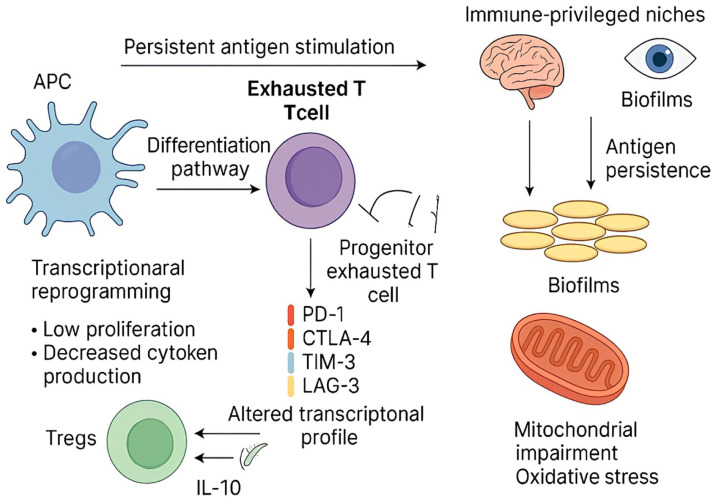
**Pathways of T-cell exhaustion in bacterial infections.** This figure illustrates the key mechanisms contributing to T-cell exhaustion during chronic bacterial infections. Persistent antigen exposure during chronic bacterial infection leads to: (i) Upregulation of inhibitory receptors: Chronic stimulation causes elevated expression of checkpoint molecules like PD-1, CTLA-4, TIM-3, and LAG-3 on CD8⁺ and CD4⁺ T cells, dampening their effector functions. (ii) Transcriptional and epigenetic reprogramming: Activation of exhaustion-associated transcription factors (e.g., TOX, NR4A) and stable epigenetic modifications enforce the exhausted phenotype. (iii) Metabolic dysregulation: Mitochondrial dysfunction and a shift from oxidative phosphorylation to glycolysis reduce the energy supply necessary for T-cell activation, further impairing immune response. (iv) Treg expansion and cytokine-mediated suppression: Regulatory T-cells (Tregs) increase in number and secrete immunosuppressive cytokines (IL-10, TGF-β), inhibiting antigen presentation and effector T-cell function. (v) Immune evasion by bacteria: Bacteria persist in immune-privileged niches (e.g., CNS, gallbladder), produce immune-modulatory molecules (e.g., Vi antigen, IL-6), and form biofilms that blunt immune recognition and clearance. APC, Antigen-Presenting Cell.

T-cell exhaustion mechanisms during bacterial infections have been identified across various pathogens (Fig. 1). Below, we summarize key mechanisms contributing to T-cell exhaustion in different bacterial infections (Table 1).

**Table 1:** Mechanisms of immune exhaustion in bacterial infections.

Infection	Dominant Exhaustion Features	Key Mechanisms	Clinical/Immunological Impact
Tuberculosis (Mtb)	↑ PD-1, CTLA-4, TIM-3 on CD4⁺/CD8⁺ T-cells ↑ Treg frequency Mitochondrial dysfunction	Chronic antigen persistence; Hypoxic granuloma microenvironment; IL-10 and TGF-β mediated suppression; Impaired oxidative phosphorylation	Reduced IFN-γ production; Poor bacterial clearance; Disease progression
Sepsis	↑ PD-1 on T cells, ↑ PD-L1 on monocytes, Lymphopenia, Reduced glucose uptake	Systemic inflammatory dysregulation; Mitochondrial dysfunction; Impaired cytokine production	Secondary infections; Increased mortality; Persistent immunosuppression
*H. pylori* infection	Treg accumulation in gastric mucosa, Suppressed Th1 responses	IL-10 mediated inhibition; Local immune tolerance induction	Chronic colonization; Persistent gastritis
Brucellosis	↑ PD-1 expression, ↑ IL-10 levels, Treg expansion	Suppressed dendritic cell activation; Reduced CD8⁺ T-cell priming	Persistent infection; Relapsing disease
Salmonellosis	↑ PD-1 and LAG-3, Reduced glycolytic enzyme expression	Antigen persistence; Impaired glycolysis (HK2, LDHA)	Decreased ATP production; T-cell functional decline
Listeriosis	↑ PD-1 expression during persistent infection, Functional CD8⁺ T-cell impairment	Chronic antigen exposure; PD-1/PD-L1–mediated inhibition; Inflammatory microenvironment stress	Reduced cytotoxic function; Delayed bacterial clearance in persistent models
Periodontitis	Increased PD-1⁺ T-cells in gingival tissue, Elevated Treg infiltration, Increased IL-10 levels	Persistent bacterial biofilm stimulation; Local immune checkpoint activation; Regulatory cytokine–mediated suppression	Chronic inflammation, Impaired bacterial clearance, Progressive tissue destruction

### Antigenic stimulation and immune checkpoint pathway activation

Immune exhaustion is often triggered by persistent antigenic stimulation. In bacterial infections, the continued presence of pathogens results in chronic activation of the immune system.^9^ This persistent activation leads to upregulation of inhibitory receptors on T-cells. These receptors suppress immune responses by inhibiting TCR signaling cascades, reducing proliferation, cytokine secretion, and cytotoxic activity, ultimately resulting in functional exhaustion.^10^

For instance, in *Mycobacterium tuberculosis* (Mtb) infections, increased expression of inhibitory receptors such as PD-1, CTLA-4, and TIM-3 on both CD4⁺ and CD8⁺ T-cells has been observed. These exhausted T-cells show reduced IFN-γ production and decreased cytotoxicity, which correlates with bacterial burden.^11^ Furthermore, transcriptomic analysis of T-cells in active Tuberculosis (TB) patients has revealed a gene expression profile akin to exhausted T-cells in chronic viral infections. PD-1 blockade in experimental human TB granuloma models partially restored T-cell cytokine secretion, directly implicating immune checkpoint signaling in functional impairment.^12^ Importantly, similar findings have been reported in human studies. In patients with active TB, increased PD-1 expression on circulating CD4⁺ and CD8⁺ T-cells has been associated with impaired IFN-γ production and reduced proliferative capacity compared to individuals with latent infection or healthy controls.^13^

Similarly, antigen persistence during *Salmonella enterica* infections has been shown to increase LAG-3 and PD-1 expression, resulting in T-cell dysfunction and bacterial persistence.^14^ These exhausted T-cells produce less IL-2 and exhibit impaired proliferative responses, highlighting the role of chronic antigen exposure in epigenetic reprogramming of T-cells, which stabilizes the exhausted phenotype.^15^ Another example is *Brucella abortus*, which is an adept at evading host immune responses by inhibiting Major Histocompatibility Complex (MHC) class II expression on monocytes/macrophages. This inhibition is mediated by Interleukin-6 (IL-6), which is induced by *Brucella* lipoproteins and suppresses IFN-γ-induced MHC-II expression.^16^

Also, studies have shown that infection with *Listeria monocytogenes* results in the upregulation of inhibitory receptors like PD-1 on antigen-specific CD8+ *T*-cells. This phenotypic alteration correlates with diminished IFN-γ production, indicating T-cell exhaustion. In a murine model of L.*monocytogenes* infection, blocking PD-1/PD-L1 signaling partially restored T-cell function and improved bacterial clearance.^17^ Moreover, elevated PD-1 expression has been correlated with disease severity and bacterial load, suggesting that checkpoint-mediated inhibition contributes to immune dysfunction in clinical settings. In septic patients, sustained upregulation of PD-1 on T-cells and PD-L1 on monocytes has also been linked to reduced cytokine production and increased susceptibility to secondary infections.^18^

In bacterial sepsis, particularly with *Pseudomonas aeruginosa* and *Staphylococcus aureus*, immune exhaustion manifests as a late-phase immune suppression phenomenon. This occurs due to widespread lymphocyte apoptosis, which triggers compensatory immune exhaustion, alongside high expression of PD-1 and CTLA-4 on T-cells, and impaired monocyte HLA-DR expression, resulting in a state of immune-paralysis.^19^ In clinical studies, increased PD-1 expression on both CD4+ and CD8+ *T*-cells in acute sepsis correlates with poor prognostic outcomes and an increased risk of subsequent infections. This heightened PD-1 expression is associated with decreased cytokine production and failure to mount an effective immune response to secondary infections.^20^ Also, septic patients exhibit decreased HLA-DR expression levels on monocytes and reduced TNF-α secretion levels following LPS stimulation, indicating both impaired antigen presentation and anergy.^21^

In patients with periodontitis, the expression of PD-1 and PD-L1 is higher than in healthy individuals, suggesting the presence of immune exhaustion in the gingival tissues. *Porphyromonas gingivalis* induces PD-L1 expression on DCs through Akt-STAT3 signaling, suppressing antigen-specific CD8+ *T*-cell responses. This suppression is manifested by decreased IFN-γ, perforin, granzyme B, and CD107a expression, leading to impaired T-cell activation and contributing to immune suppression in the periodontal environment.^22^

### Imbalance between effector and regulatory T-cells

In bacterial infections, an imbalance between effector T-cells and regulatory T-cells (Tregs) can exacerbate immune exhaustion. For example, in chronic infections like TB, an expansion of Tregs has been observed, which correlates with poor bacterial clearance and prolonged infection, suggesting that Tregs play a role in suppressing protective immune responses.^23^ Tregs exert their suppressive function through cytokines such as Interleukin-10 (IL-10) and Transforming Growth Factor-Beta (TGF-β). IL-10 inhibits antigen presentation and cytokine production, while TGF-β suppresses T-cell proliferation and differentiation.^24^

Importantly, evidence from human studies supports this mechanism. Increased frequencies of circulating and pulmonary FoxP3⁺ Tregs have been reported in patients with active TB compared to latent infection or healthy controls, and their expansion has been associated with reduced Th1 responses and impaired IFN-γ production.^23^ Elevated IL-10 levels in TB patients have also been linked to diminished macrophage activation and persistent infection,^25^ reinforcing the role of regulatory pathways in human disease.

Studies in humans infected with *Salmonella* have shown that T-cell exhaustion is a major contributor to the inability to clear the pathogen effectively. For example, in the liver, *Salmonella* persistence is linked to the presence of immunoregulatory CD4+ *T*-cells and alternatively activated macrophages. These immune cells secrete anti-inflammatory cytokines such as IL-10, which impair the bactericidal activity of immune cells, thereby supporting bacterial survival.^26^ Furthermore, Salmonella infection leads to an increase in PD-L1 and TGF-β expression in Kupffer cells (liver macrophages), contributing to an exhausted and tolerogenic immune state.^27^ Also, In the gallbladder, *Salmonella* forms biofilms on gallstones, which create a persistent environment that favors bacterial survival. This niche promotes a shift in the immune response from a proinflammatory Th1 response to an anti-inflammatory Th2 response. The Th2 response is marked by increased immunoglobulin production and the Th2 transcription factor GATA3, contributing to immune tolerance and bacterial persistence.^7^

*Brucella* infection leads to the upregulation of inhibitory immune checkpoint receptors, particularly PD-1, on Tregs. The expression of PD-1 and Glucocorticoid-Induced Tumor necrosis factor Receptor (GITR) is elevated in both acute and chronic brucellosis, while CTLA-4 expression is elevated specifically in chronic cases. This overexpression of immune checkpoints on Tregs contributes to immune suppression and may facilitate the persistence of *Brucella* infection.^28^ In *Brucella abortus* infection, Treg expansion is accompanied by increased IL-10 production, which inhibits dendritic cell activation and CD8⁺ T-cell priming.^16^ Consistently, patients with chronic brucellosis have demonstrated elevated IL-10 levels and regulatory T-cell activity, correlating with persistent infection.^29^ Moreover, murine models have shown that *Brucella* infection upregulates IL-10 production by both Tregs and infected macrophages, dampening the Th1-type cytokine response. This creates an immune environment that favors pathogen persistence by reducing macrophage activation.^30^

Studies of *Helicobacter pylori* infections have shown a similar trend, with Treg accumulation in the gastric mucosa inhibiting the development of effective Th1 responses, promoting chronic colonization.^31^ In human *H. pylori* infection, increased gastric mucosal Treg infiltration has been associated with higher bacterial density and suppressed local inflammatory responses.^32^

In murine models of *Streptococcus pneumoniae* colonization, Treg depletion enhances bacterial clearance, further supporting the role of Tregs in regulating immune exhaustion.^33^ Furthermore, *P. gingivalis* Lipopolysaccharide (LPS) activates TLR2 and TLR4 on DCs, leading to the production of proinflammatory cytokines such as IL-1β and IL-10. This activation is associated with inflammasome activation and sustained cytokine release, contributing to chronic inflammation in periodontal tissues.^34^

### Metabolic dysfunction in exhausted T-cells

Metabolic dysfunction in exhausted T-cells plays a critical role in their impairment. In chronic bacterial infections, the metabolic demands of T-cells are altered, with exhausted cells exhibiting impaired mitochondrial function and reduced glycolytic activity. Deficiencies in pathways such as oxidative phosphorylation and fatty acid metabolism restrict energy availability, limiting T-cell activation and survival. These metabolic disruptions hinder T-cell function, contributing to immune failure in chronic bacterial infections.^35^

For example, in Mtb infection cases, CD8⁺ T-cells within granulomas show evidence of mitochondrial depolarization and bioenergetic exhaustion. This exhaustion is aggravated by competition for nutrients in the hypoxic granuloma environment.^36^ Importantly, emerging human data support these observations. In patients with active TB, altered mitochondrial function and reduced bioenergetic capacity have been reported in circulating T-cells, with impaired oxidative phosphorylation correlating with diminished effector cytokine production.^37^ Metabolic profiling studies have also demonstrated systemic metabolic reprogramming in TB patients, including disruptions in lipid metabolism and energy pathways that may indirectly affect T-cell fitness.^38^ In septic patients, circulating T-cells exhibit reduced mitochondrial respiration and decreased ATP production, consistent with functional immunosuppression and poor clinical outcomes.^39^ Also, Mtb manipulates host macrophage responses by inducing an M2-like polarization state, which is associated with anti-inflammatory activity and tissue repair rather than microbial killing. This further impairs antigen presentation and cytokine production, undermining effective immunity and promoting granuloma stability.^40^^,^^41^ Additionally, Mtb secretes molecules such as ESAT-6, which affect MHC-II expression and antigen processing in dendritic cells, limiting T-cell priming and promoting immune evasion.^42^

Similarly, in chronic *Salmonella* infection models, exhausted T-cells exhibit reduced expression of key glycolytic enzymes, such as HK2 and LDHA, leading to diminished ATP production and impaired effector function.^1^ Furthermore, studies have demonstrated that L.*monocytogenes* can alter host lipid metabolism in infected macrophages, leading to impaired antigen presentation and reduced expression of co-stimulatory molecules like CD80 and CD86, which are crucial for T-cell activation and survival.^43^

Recent studies on sepsis-induced bacterial infections also highlight reduced glucose uptake and fatty acid oxidation in circulating T-cells, further reinforcing the critical role of metabolic dysfunction in immune exhaustion.^44^ Moreover T-cell dysfunction, the expansion of Myeloid-Derived Suppressor Cells (MDSCs) plays a significant role in immune dysfunction during sepsis. MDSCs contribute to immune exhaustion by releasing arginase-1, Reactive Oxygen Species (ROS), and IL-10, all of which inhibit T-cell responses and promote apoptosis.^34^ Recent single-cell RNA sequencing studies of blood samples from septic patients have shown widespread transcriptional upregulation of exhaustion-associated genes, such as TOX, EOMES, and LAG3, across both adaptive and innate immune compartments. These findings highlight the systemic nature of immune exhaustion during sepsis, affecting multiple layers of the immune system.^45^

## Difference mechanistic features of bacterial and viral T-cell exhaustion

Chronic viral infections, like HIV and hepatitis, can lead to T-cell exhaustion driven by high or persistent levels of viral antigens. Exhausted T-cells in these viral situations express high amounts of inhibitory molecules, such as PD-1, CTLA-4, LAG-3, and TIM-3, and often make less of the cytokines IL-2 and TNF than functional T-cells. When PD-1 and other checkpoints are expressed, they are good markers of exhaustion and can be used as targets for checkpoint blockade therapy to restore some function in these T-cells during chronic viral infection.^2^

Conversely, in bacterial infections, T-cell exhaustion is driven by repeated exposure to bacterial antigens and overstimulation of T-cells due to chronic inflammation, but this process may not follow the same mechanisms as that of viral infections. In patients with Common Variable Immunodeficiency (CVID), who are prone to recurrent bacterial infections, the CD4+ *T*-cells making an immune response to bacterial antigens have a higher expression of PD-1 and exhibit more functional impairment than those to viral antigens from the same patient. Thus, the continuous stimulation of T-cells by bacterial products, such as during bacterial translocation or mucosal leakage, may contribute to the greater production of exhausted T-cells specific for bacterial antigens.^46^^,^^47^

A key difference between bacterial and viral exhaustion mechanisms is the presence of inhibiting receptors (like Tim-3) during chronic Mtb infection that are present on Tim-3 + *T*-cells after they co-express exhaustion markers, demonstrate reduced IL-2 and TNF production, and show increased production of IL-10 as a cytokine that plays an important role in providing immunosuppressive feedback. The combination of the cytokine environment and the co-expression of receptors indicate the presence of a complex array of signal events of suppressive-type signals which differ from the context of viruses, where IFN-γ dysregulation and persistent viral replication are more pronounced.^9^

A major mechanistic distinction exists between bacterial and viral exhaustion, based on the patterns of antigen persistence and the inflammatory context. The persistence of viruses is normally due to the mechanisms that both replicate the virus over time and help the virus evade the immune response. Thus, this leads to a virus being continuously present in the host and associated with ongoing exposure to viral peptides, ultimately resulting in terminal exhaustion. Contrarily, at the time of bacterial antigen exposure, key mechanisms contributing to the continued exposure include continued inflammation, translocation at a subclinical level, and the presence of impaired barrier function (e.g., gut or lung mucosa), which could result in a more restricted and, potentially, reversible type of exhaustion in T-cells that are specific to bacterial antigens.^48^

Finally, translational relevance for both contexts must consider species differences. Although there are substantial amounts of human data regarding the use of therapeutic blockade against the immune checkpoints in viral infections and clinical studies of checkpoint blockade for cancer, there is a rising amount of human data regarding bacterial T-cell exhaustion; however, there are fewer total human data regarding bacterial T-cell exhaustion than concerning viral T-cell exhaustion. The recognized need for additional clinical studies to elucidate our mechanistic understanding from both human cohorts and animal models demonstrates this point.^49^

## Impact of immune exhaustion on clinical outcomes

### Chronic infections and persistent pathogens

In bacterial infections like TB, immune exhaustion contributes to the failure of the immune system to clear pathogens, leading to chronic infection and tissue damage. For example, Mtb can survive within immune cells for extended periods, evading clearance and exacerbating immune exhaustion. This persistent infection creates a cycle of inflammation, tissue damage, and immune suppression, ultimately impairing the body’s ability to control the infection.^50^

A similar pattern is observed in chronic *Salmonella* carriers, where exhausted T-cell populations in the gallbladder and liver permit the pathogen to persist without causing clinical symptoms, while periodically shedding bacteria and transmitting the infection to new hosts. In chronic infections caused by *Brucella* or *P. gingivalis*, T-cell exhaustion prevents macrophages from activating fully, allowing the pathogens to survive in immune-privileged niches during chronic inflammation.^51^

These examples highlight how T-cell exhaustion impedes sterilizing immunity, enabling pathogens to coexist with the host in a state of immune tolerance. In human patients, this can manifest as chronic, recurrent, or persistent infections, where the immune system is unable to effectively combat the pathogen. As a result, these patients may experience prolonged illness and complications that arise from sustained infection.

### Increased susceptibility to secondary infections

Immune exhaustion not only impacts the primary infection but also increases susceptibility to secondary infections. In sepsis, for example, immune exhaustion leads to impaired responses to subsequent infections, contributing to high mortality rates. Studies have shown that septic patients often exhibit prolonged PD-1 expression on CD8+ *T*-cells, which is associated with a higher risk of nosocomial infections, such as *Pseudomonas, Candida*, and *Acinetobacter*. This sustained immune dysfunction complicates the clinical management of these patients.^52^

In patients recovering from sepsis or other critical illnesses, immune exhaustion can lead to prolonged periods of immune suppression, leaving individuals vulnerable to secondary infections. Research has demonstrated that T-cell exhaustion impairs the ability to mount an effective response to new microbial challenges, leading to an increased burden of secondary infections in hospital settings.^53^ These findings underscore the importance of addressing immune exhaustion in clinical settings, particularly in patients who have survived severe infections.

### Antibiotic resistance

Immune exhaustion significantly contributes to the emergence of antibiotic resistance through several mechanisms. In immunocompromised or immune-exhausted individuals, reduced immune-mediated pathogen clearance enables prolonged bacterial survival under antibiotic pressure, which increases the likelihood of resistance-conferring mutations. For example, in infections like chronic osteomyelitis, immune exhaustion impairs effective bacterial clearance, allowing for prolonged sub-inhibitory antibiotic exposure and promoting the selection of resistant strains.^54^

In human patients with chronic infections, such as those caused by *S. aureus*, immune exhaustion is associated with persistent infections despite the use of antibiotics. In murine models of osteomyelitis, the combination of reduced T-cell function and elevated IL-10 levels contributed to persistent *S. aureus* infections, which could not be eradicated even with antibiotics.^55^^,^^56^ These findings suggest that immune exhaustion may facilitate the development of antibiotic resistance, as the immune system’s inability to clear pathogens allows bacteria to survive and evolve in the presence of antibiotics.^57^

### Microbiome modulation

Immune exhaustion profoundly influences the gut microbiota. This bidirectional relationship between immune function and microbiota composition has significant implications for host health, particularly in the context of chronic diseases and therapeutic interventions. Chronic immune activation, as seen in bacterial infections, can disrupt the delicate balance of the gut microbiota, leading to dysbiosis. This imbalance favors the proliferation of pathogenic bacteria over beneficial species, compromising gut barrier integrity and intensifying systemic inflammation.^58^

In human patients with chronic inflammatory conditions, immune exhaustion can exacerbate microbiome dysbiosis, further impairing immune responses. For example, dysbiosis in patients with chronic *Clostridium difficile* infections has been shown to worsen disease severity and prolong recovery times. This disrupted microbiota composition may also impact the effectiveness of immunotherapies, such as immune checkpoint inhibitors.^59^ Interestingly, recent studies have shown that modulating the microbiota through dietary interventions or probiotics can help restore immune function, reduce inflammation, and improve outcomes in patients suffering from chronic infections.^60^

The depletion of butyrate-producing bacteria, such as *Faecalibacterium prausnitzii*, has been shown in murine models to cause increased intestinal permeability and higher levels of systemic IL-6 and TNF-α, both of which promote T-cell exhaustion. Conversely, restoring beneficial microbes like *F. prausnitzii* through probiotic supplementation can help reverse T-cell exhaustion and improve immune function in the gut and beyond.^61^

## Therapeutic strategies to reverse T-cell exhaustion

### Immune checkpoint inhibitors (ICIs)

Immune Checpoint Inhibitors (ICIs) are agents that target inhibitory receptors such as PD-1, CTLA-4, and others, to reinvigorate exhausted T-cells and restore their effector functions. These therapies have shown promise in cancer immunotherapy, and recent studies suggest that they may also be effective in bacterial infections. However, their effectiveness is often limited to progenitor exhausted T-cells, with terminally exhausted T-cells exhibiting resistance to reactivation.^22^

In bacterial sepsis models in mice, PD-1 inhibition has been shown to improve survival and resistance to secondary infections, suggesting that ICIs could be a promising therapeutic avenue for reversing immune exhaustion in bacterial infections.^62^ In human clinical trials, the use of anti-PD-1 therapy in patients with chronic infections such as TB has demonstrated partial restoration of T-cell function and cytokine secretion, although the response is often suboptimal in those with severe immune exhaustion.^63^

ICIs are also being investigated in the context of chronic *Salmonella* infections. Studies have shown that PD-1 blockade can improve T-cell responses and bacterial clearance in mouse models of *Salmonella typhimurium* infection.^64^ These findings suggest that immune checkpoint inhibition could help reverse T-cell exhaustion in chronic bacterial infections, potentially improving patient outcomes.

### Combination therapies

Combining ICIs with other treatments, such as 4–1BB agonists, epigenetic modulators, metabolic reprogramming agents, and cytokine therapies (e.g., IL-2), has shown enhanced efficacy in preclinical models. By targeting multiple facets of T-cell exhaustion, these combination therapies have the potential to restore immune function more effectively than monotherapies.^65^ For instance, co-administration of PD-1 inhibitors with 4–1BB agonists has been shown to increase mitochondrial biogenesis and cytokine production in both acute and chronic bacterial infections, as well as in chronic viral infections.^66^

In recent studies on humans, methods combining immunomodulators to treat severe infections that resistance to treatment such as persistent, chronic illnesses are being researched. Studies using ex-vivo samples from septic patients with combined inhibition of PD-1 and PD-L1 showing greater improvement in T-cell proliferation and cytokine production when the two methods were used together than if either were used alone.^67^ The use of the cytokine IL-7 in critically ill septic patients significantly reduced lymphopenia and enhanced T-cell function, indicating that IL-7 should be included as part of all combination techniques with the goal of restoring immunity.^68^

In pulmonary tuberculosis, there have been limited investigations of host-directed therapies using immune checkpoints in the treatment of AIDS-related tuberculosis in combination with either metabolic-based or cytokine-based therapies. The use of immune checkpoint inhibitors is not part of the standard treatment of tuberculosis; however, there is evidence in cancer patients who received PD-1 inhibitors and experience immune reactivation against latent Mtb. This finding shows that the modulation of immune checkpoints in human patients has the potential to significantly alter immune response against mycobacterial host cell infection.^69^ These information reflect the new developments related to restoring the immune response against chronic bacterial infections, as well as indicate the need for meticulous immune balancing in multiple treatment regimens of patients presenting with bacterial infection.

### Mitochondrial targeting

Strategies aimed at restoring mitochondrial function, including enhancing oxidative phosphorylation and reducing Reactive Oxygen Species (ROS), have demonstrated potential in reversing T-cell exhaustion. Mitochondrial uncouplers or PGC1α activators, when used in exhausted T-cells, increase ATP levels, cytokine secretion, and responsiveness to antigenic stimulation.^62^ These strategies are particularly promising for improving immune responses in chronic bacterial infections, where T-cell exhaustion is closely linked to mitochondrial dysfunction and bioenergetic exhaustion.

In mouse models of Mtb infection, restoring mitochondrial function has been shown to reinvigorate exhausted T-cells, improving their capacity to clear the infection.^3^ Similarly, in chronic *Salmonella* infection, metabolic reprogramming through mitochondrial modulation has been found to enhance T-cell effector function and bacterial clearance.^70^ These findings suggest that targeting mitochondrial dysfunction could be a viable therapeutic strategy to combat immune exhaustion in chronic bacterial infections.

### Chimeric antigen receptor (CAR) T-cell engineering

Chimeric Antigen Receptor (CAR) T-cells are genetically modified T-cells that express receptors specific to a target antigen, enhancing their ability to recognize and attack infected cells. CAR T-cell therapy has shown great success in cancer treatment, and recent research is exploring its potential in bacterial infections. Modifying CAR T-cells to resist exhaustion through intermittent signaling or genetic alterations can enhance their persistence and effector function.^71^

In experimental models of chronic bacterial infections, such as L.*monocytogenes*, CAR T-cells engineered to resist exhaustion have shown improved bacterial clearance and reduced tissue damage.^72^ While CAR T-cell therapy for bacterial infections is still in its early stages, these studies suggest that it holds promise as an innovative approach to treat chronic infections that involve T-cell exhaustion.

### Modulation of the microbiota

Interventions such as Fecal Microbiota Transplantation (FMT), probiotics, and prebiotics have shown potential in restoring microbial balance and enhancing immune function. FMT has been found to improve responses to immune checkpoint inhibitors in cancer therapy by modulating the gut microbiome.^73^ Additionally, dietary interventions that increase fiber intake have been shown to enrich beneficial microbial populations, supporting immune homeostasis.^74^ Recent studies suggest that certain bacterial metabolites, such as indole derivatives and bile acids, can directly modulate T-cell exhaustion markers.^75^

In patients with chronic infections, such as *C. difficile* or *H. pylori*, restoring a healthy microbiota through probiotic supplementation has been associated with improved immune function and reduced immune exhaustion. For example, FMT from ICI-responsive donors has been shown to increase CD8+ *T*-cell activity and decrease PD-1 expression in both infection and cancer preclinical models.^74^^,^^76^ These findings highlight the potential of microbiota modulation as a therapeutic strategy to reverse immune exhaustion in chronic bacterial infections and improve patient outcomes.

## Conclusion

**Fig. 2:**
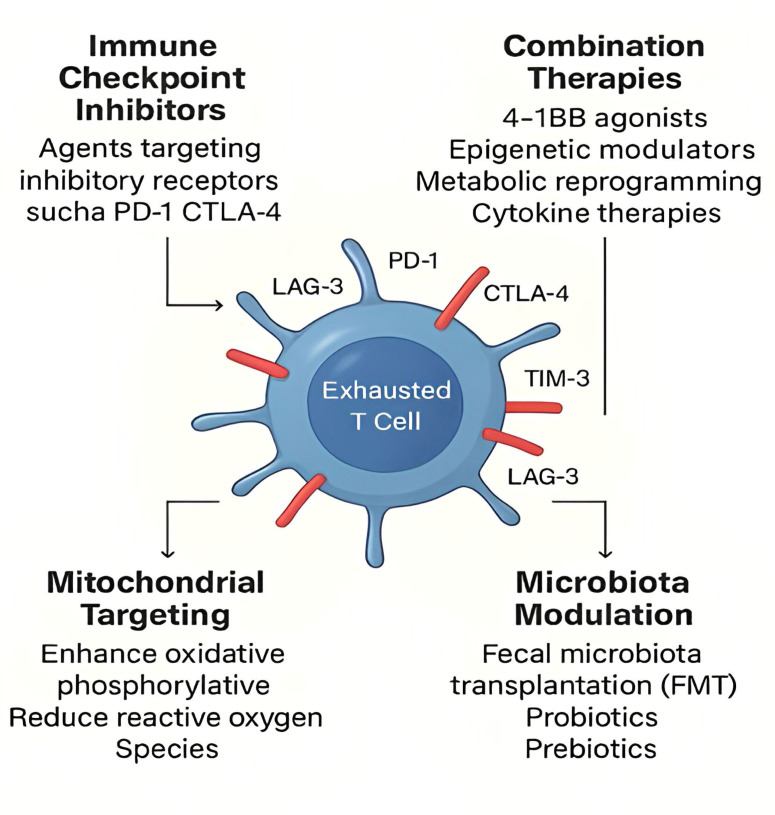
**Therapeutic strategies to reverse T-cell exhaustion.** This figure illustrates key therapeutic strategies designed to counteract T-cell exhaustion in chronic bacterial infections: (i) Immune Checkpoint Inhibitors (ICIs): Blockade of inhibitory receptors such as PD-1 and CTLA-4 can reinvigorate exhausted T-cells and restore their effector functions, particularly in progenitor-exhausted subsets. (ii) Combination therapies: The integration of ICIs with agents like 4–1BB agonists, epigenetic modulators, cytokines (e.g., IL-2), and metabolic reprogramming compounds enhances therapeutic outcomes by addressing multiple exhaustion pathways. (iii) Mitochondrial targeting: Approaches aimed at restoring mitochondrial function include promoting oxidative phosphorylation, enhancing fatty acid oxidation, and reducing reactive oxygen species to improve T-cell metabolism and function. (iv) Microbiota modulation: Interventions such as Fecal Microbiota Transplantation (FMT), prebiotics, probiotics, and dietary fiber aim to rebalance gut microbiota, enhancing systemic immunity and reversing immune dysfunction associated with T-cell exhaustion.

Immune exhaustion plays a crucial role in chronic bacterial infections by impairing pathogen clearance and promoting infection persistence. Persistent antigen stimulation, upregulation of inhibitory receptors, expansion of regulatory T-cells (Tregs), and metabolic dysfunction are key factors contributing to T-cell exhaustion during chronic bacterial infections. The consequences of immune exhaustion include impaired bacterial clearance, increased susceptibility to secondary infections, antibiotic resistance, and microbiome disruption. Given the complexity of immune exhaustion and its impact on chronic infections, continued research is essential to fully elucidate the mechanisms involved and to optimize therapeutic interventions. By targeting immune exhaustion, it may be possible to not only improve outcomes for individuals with chronic bacterial infections but also enhance the efficacy of existing treatments and reduce the burden of antibiotic resistance.(Fig. 2)

## Declaration of generative AI and AI-assisted technologies

AI tools were used solely for enhancing the language, grammar, and overall writing quality of this manuscript. The authors confirm that these tools were not used for generating data, conducting analyses, or fabricating any part of the research content.

## Data availability

The data that support the findings of this study are available from the corresponding author upon reasonable request.

## Conflicts of interest

The authors declare no conflicts of interest.
